# Association Between Retinol-Binding Protein 4 Levels and Hepatitis C Virus Infection: A Meta-Analysis

**DOI:** 10.3390/diseases12110291

**Published:** 2024-11-13

**Authors:** Yingying Lin, Xinyu Cui, Na Zhu, Yanyan Li, Peng Wang, Xin Wang, Yunyun Yi, Xin Li

**Affiliations:** 1 Department of Center of Integrated Traditional Chinese and Western Medicine, Peking University Ditan Teaching Hospital, No. 8, Jingshun East Street, Chaoyang District, Beijing 100015, China; linyingying0123@163.com (Y.L.); zzuwxin@163.com (X.W.); 2 Department of Center of Integrated Traditional Chinese and Western Medicine, Beijing Ditan Hospital, Capital Medical University, No. 8, Jingshun East Street, Chaoyang District, Beijing 100015, China; cxy13373145868@163.com (X.C.); zhuna202203@163.com (N.Z.); 15736990162@163.com (Y.L.); wpeng0927@163.com (P.W.); yyygrace@126.com (Y.Y.)

**Keywords:** retinol-binding protein 4, hepatitis C virus, liver function, human, meta-analysis

## Abstract

*Background and Objectives*: The relationship between circulating retinol-binding protein 4 (RBP4) levels and hepatitis C virus (HCV) infection remains unclear. This study aims to systematically assess RBP4 expression in patients with HCV and its correlation with disease severity. *Materials and Methods*: We searched the Embase, PubMed, and Cochrane databases for relevant studies up to 1 January 2024. This study was registered on PROSPERO (CRD42023489051). *Results*: Our analysis included eight studies with 2612 participants (1152 controls and 1282 patients with HCV). Overall, RBP4 levels did not significantly differ between patients with HCV and controls (SMD: −0.36; 95% CI: −0.94, 0.23; *p* = 0.23). However, in a subgroup of Asian subjects, patients with HCV showed significantly lower RBP4 levels (SMD: −0.40; 95% CI: −0.49, −0.31; *p* = 0.10). Additionally, a negative correlation between RBP4 levels and disease severity was observed across all studied populations. *Conclusions*: RBP4 levels may vary due to HCV genotype, ethnicity, and environmental factors. In the context of HCV infection, RBP4 levels appear to reflect the severity of disease progression. Our findings indicate that RBP4 could serve as a biomarker for HCV disease progression. Further research is needed to elucidate the complex mechanisms of RBP4 in HCV infection.

## 1. Introduction

Globally, approximately 71.1 million individuals suffer from chronic hepatitis C virus (HCV) infection [1]. HCV is classified into 7 genotypes and 67 subtypes, with variations in regional and ethnic distribution. These genotypes exhibit distinct clinical profiles and treatment responses [2]. HCV can affect human metabolism, with well-documented links to glucose and lipid metabolism disruptions [3,4,5]. Consequently, patients with HCV are not only at risk for liver-related complications such as cirrhosis and hepatocellular carcinoma but also extrahepatic metabolic disorders, including insulin resistance and diabetes mellitus [6,7].

Retinol-binding protein 4 (RBP4), an adipocytokine in the lipid-transporting protein family [8], is the only carrier of retinol in the bloodstream [9]. Hepatocytes are the primary source of RBP4, with adipose tissue also contributing to its synthesis [10]. Beyond its established role in lipid metabolism, RBP4 is increasingly recognized for its association with insulin resistance [11,12]. Tsutsumi et al. have proposed that metabolic alterations in HCV infection may be related to RBP4 [13]. Growing evidence supports the role of RBP4 in HCV pathogenesis and suggests a potential link between RBP4 levels and metabolic dysregulations in HCV infection [14,15].

However, the relationship between HCV infection and circulating RBP4 levels remains inconsistent across studies. Some reported elevated RBP4 levels in patients with HCV [16,17], while others found decreased levels [18,19,20,21,22] or no significant correlation with HCV status [23]. Given this controversy, a systematic evaluation and meta-analysis are urgently needed to clarify the relationship between RBP4 levels and HCV infection.

## 2. Materials and Methods

This meta-analysis adheres to the Preferred Reporting Items for Systematic Reviews and Meta-analyses (PRISMA) guidelines [24], and is registered prospectively on Prospero (International Prospective Register of Systematic Reviews—University of York) (ID: CRD42023489051).

### 2.1. Search Strategy and Study Selection

Two reviewers (Yingying Lin and Xinyu Cui) independently conducted a literature search of the Embase, PubMed, and Cochrane databases from inception to 1 January 2024, following a predefined strategy. The search terms included (“retinol binding protein 4” OR RBP4) AND (“hepatitis C” OR “hepatitis C virus “ OR “hepatitis non A non B” OR HCV). The search criteria were not limited by languages, geographical regions, or study designs. Furthermore, the reference lists of relevant articles were meticulously scrutinized to identify any additional eligible studies.

### 2.2. Inclusion and Exclusion Criteria

All studies reporting circulating RBP4 levels in patients with HCV were eligible for this review. Other inclusion criteria included: (1) age ≥ 18 years old; (2) observational studies involving humans; (3) studies containing the measurement results of circulating RBP4 levels both in patients with HCV and healthy people.

Exclusion criteria: (1) studies involving patients with other liver disease, systemic conditions, or comorbidities, including hepatitis B virus, schistosomiasis, autoimmune or alcoholic hepatitis, diabetes, hepatocellular carcinoma, and acquired immunodeficiency syndrome; (2) studies that have been intervened at baseline; (3) the population of the study overlapped with another study; (4) reviews, letters, conference abstracts, case reports, or animal or cell line studies.

### 2.3. Data Extraction

Data extraction was conducted independently by two researchers (Yingying Lin and Xinyu Cui) using a specially designed Excel sheet. Discrepancies were resolved by discussing with the third researcher (Na Zhu). We contacted corresponding authors for further information via email when data were incomplete. If the corresponding authors failed to respond or were unable to provide the necessary data, we attempted data conversion using standard formulas. Studies lacking essential data were excluded. The extracted data included study characteristics: first author, publication year, country, study design type, and RBP4 measurement method.

### 2.4. Quality Assessment

The Newcastle-Ottawa Quality Assessment Scale (NOS) [25], a widely recognized instrument, was employed to evaluate the quality of included studies. The scoring system assigns an overall quality score from 0 to 9, classifying the study as low (0–3), moderate (4–6), or high (7–9) quality.

### 2.5. Statistical Analyses

Analyses were performed using Review Manager (RevMan, Version 5.4.1) and R Studio (Version 4.2.3) with the “meta” package. A two-sided *p*-value < 0.05 was considered statistically significant. Effect sizes were estimated using standardized mean differences (SMDs) with 95% confidence intervals (CIs). Correlation coefficients (r) were transformed to Z-values using Fisher’s Z-transform. The aggregated Z-values were converted back to r for assessing associations between clinical parameters and RBP4 levels. Correlation strengths were interpreted as weak (0.1–0.3), moderate (0.3–0.5), or strong (0.5–1.0).

Heterogeneity was evaluated using the Chi^2^ test and the I^2^ test. Substantial heterogeneity was defined as *p* < 0.1 or I^2^ > 50%. Random-effect models were used in the presence of significant heterogeneity; otherwise, fixed-effect models were applied. Subgroup analyses by continent were conducted to explore sources of heterogeneity. Sensitivity analysis was performed by sequentially omitting one study at a time and recalculating the pooled SMD. Publication bias was investigated by the funnel plot and Egger’s test. Additionally, a “trim and fill” analysis was conducted to adjust for potential publication bias.

## 3. Results

### 3.1. Literature Selection

**Figure 1:**
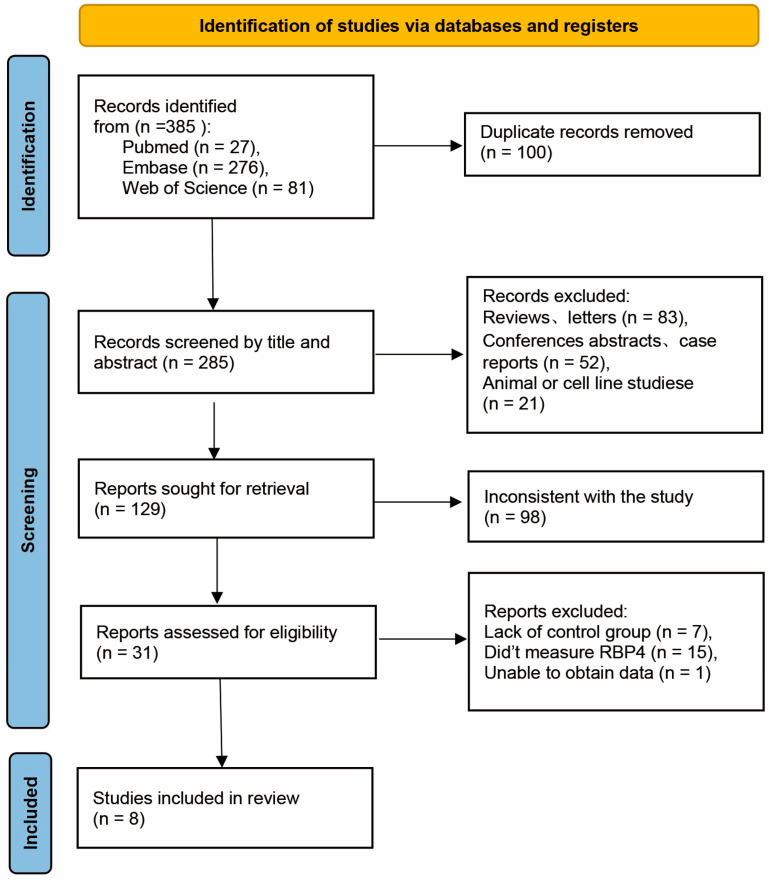
Flow chart showing the study selection process.

The flowchart in Figure 1 summarizes the process of study identification, screening, and final selection for this meta-analysis. We initially retrieved 385 articles, which were narrowed down to 31 after duplicate removal and relevance assessment. Full-text review led to the exclusion of 23 articles, leaving 8 studies for the final analysis. Two of these studies compared RBP4 levels in patients with HCV with and without steatosis, three stratified patients with HCV by fibrosis stage [no, mild (F0–F2), and severe (F3–F4)], and four reported the correlation between HCV RNA levels and RBP4 concentrations. Data on the correlation between fibrosis staging and RBP4 levels were available in two studies. Two studies provided data on the relationship between insulin resistance and RBP4 levels, and three studies offered correlation coefficients for the relationship between aspartate aminotransferase (AST) and alanine aminotransferase (ALT) levels with RBP4 levels.

### 3.2. Characteristics of the Included Studies

Table 1 details the main characteristics of the included studies, published between 2009 and 2023, which included a total of 2434 subjects (1152 controls and 1282 patients with HCV). Geographically, the studies were from Asia (*n* = 3), Europe (*n* = 3), and Africa (*n* = 2). All were observational, consisting of one cohort, one case-control, and six cross-sectional studies. Peripheral blood RBP4 levels were measured using enzyme-linked immunosorbent assay (ELISA) in all studies.

### 3.3. Quality of Included Studies

Using the modified NOS, we categorized two studies as low quality, five as medium quality, and one as high quality. No study was excluded because of a poor NOS score (≤2).

### 3.4. Meta-Analysis

**Figure 2:**
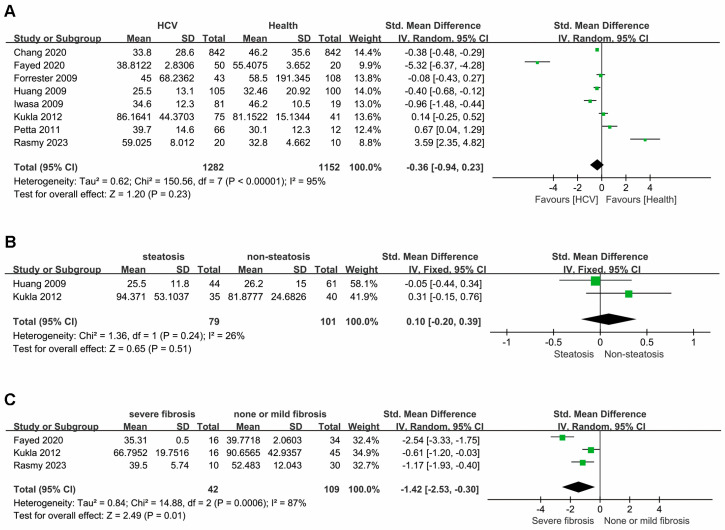
Forest plot of RBP4 levels: (**A**) patients with HCV and health controls; (**B**) patients with HCV with steatosis and without steatosis; (**C**) patients with HCV with severe fibrosis and patients with HCV with no or mild fibrosis.

A random-effects model was used for the meta-analysis of the eight extracted studies due to significant heterogeneity (I^2^ = 95%; *p* < 0.01) (Figure 2). The pooled analysis indicated no significant difference in RBP4 levels between patients with HCV and healthy controls (SMD: −0.36; 95% CI: −0.94, 0.23; *p* = 0.23). Similarly, no significant difference was observed in RBP4 levels between patients with HCV with (*n* = 79) and without steatosis (*n* = 101) (SMD: 0.10; 95% CI: −0.20 to 0.39; *p* = 0.51). However, RBP4 levels were significantly lower in patients with severe liver fibrosis (*n* = 42) than in those with no or mild fibrosis (*n* = 109) (SMD: −1.42; 95% CI: −2.53, −0.30; *p* = 0.01), suggesting a potential association between RBP4 and liver fibrosis in patients with HCV.

**Figure 3:**
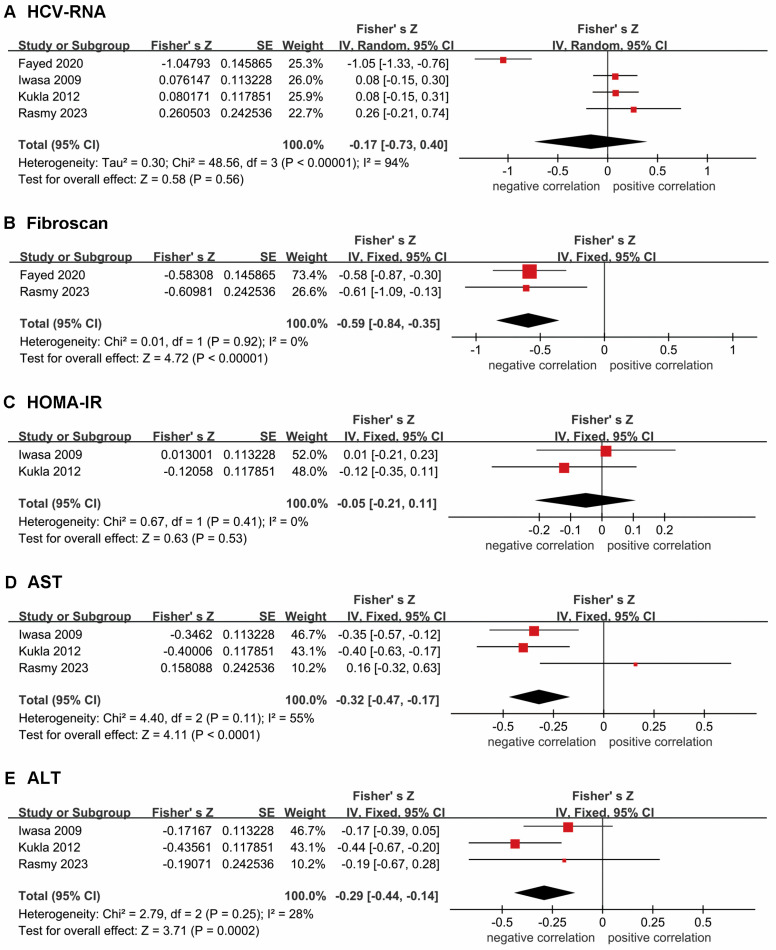
Forest plot of the correlation between RBP4 and some clinical parameters: (**A**) HCV-RNA levels; (**B**) fibrosis degree measurements by fibroScan; (**C**) HOMA-IR; (**D**) AST; (**E**) ALT.

To further explore the correlation between disease progression and RBP4 levels in patients with HCV, we assessed the r between circulating RBP4 levels and several clinical parameters, including HCV RNA levels, fibrosis measurements, homeostasis model assessment for insulin resistance (HOMA-IR), and levels of AST and ALT (Figure 3). RBP4 was negatively correlated with hepatic fibrosis severity, and with AST and ALT levels. However, no significant association was observed with HCV RNA levels or HOMA-IR. The combined Fisher’s Z values were subsequently converted to r (Table 2). The analysis showed a weak negative correlation between AST levels and RBP4 levels (r = −0.29; 95% CI: −0.41, −0.17), moderate negative correlation with ALT levels (r = −0.31; 95% CI: −0.44, −0.17), and a strong negative correlation with the degree of fibrosis (r = −0.53; 95% CI: −0.69, −0.37).

### 3.5. Subgroup Analysis

**Figure 4:**
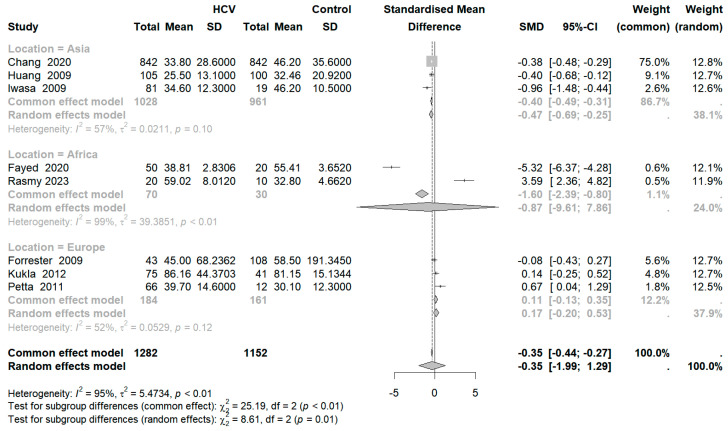
Subgroup analysis by continent.

To examine whether regional differences could account for the observed high heterogeneity, we performed subgroup analyses categorized by continent (Asia, Africa, Europe). As shown in Figure 4, the Asian subgroup (I^2^ = 57%; *p* = 0.01) and the European subgroup (I^2^ = 52%; *p* = 0.12) exhibited lower heterogeneity, thus meeting the criteria for fixed-effects modeling, whereas the African subgroup maintained high heterogeneity (I^2^ = 99%; *p* < 0.01). Notably, RBP4 levels were significantly lower in Asian patients with HCV compared to healthy controls (SMD: −0.40; 95% CI: −0.49, −0.31), while no significant differences were observed within the European and African cohorts.

### 3.6. Sensitivity Analysis

**Figure 5:**
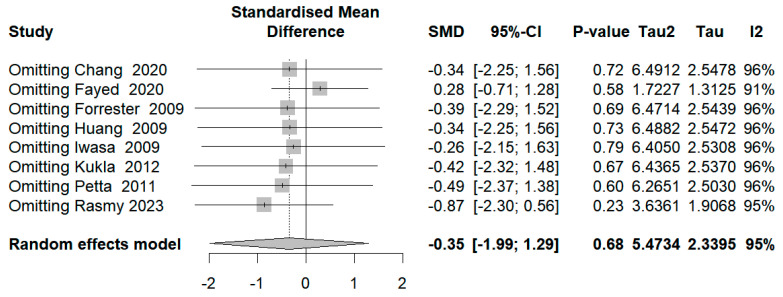
Sensitivity analysis plot.

Sensitivity analyses utilizing the “leave-one-out” method demonstrated the stability of our findings, with stable direction and magnitude of the pooled estimates following sequential omission of individual studies. It suggests that no single study disproportionately influenced the results (Figure 5).

### 3.7. Publication Bias

Visual inspection of funnel plots suggested potential asymmetry. To mitigate the subjectivity inherent in such interpretation, an Egger’s test was conducted, showing no significant evidence of publication bias (*p* = 0.1964) (Supplementary Figure S1). The Duval and Tweedie trim and fill technique did not substantially alter the comparison of RBP4 levels between patients with HCV and healthy controls, reinforcing the reliability of our findings (Supplementary Figure S2).

## 4. Discussion

RBP4, a critical metabolic molecule, plays a role in various physiological processes, including inflammatory responses, lipid metabolism, and tissue repair [26,27,28]. Its levels can be influenced by metabolic status and liver function, both of which may be compromised in HCV infection [29]. This complexity has contributed to the conflicting findings regarding RBP4 levels in patients with HCV. A meta-analysis focused on HCV infection and RBP4 levels is therefore warranted to synthesize available data and to guide the trajectory of future research endeavors. To our knowledge, this study is the first to explore the correlation between RBP4 levels and HCV infection, aiming to synthesize current knowledge and inform future research directions.

Our findings did not establish a clear correlation between circulating RBP4 levels and HCV infection, prompting subgroup analyses to identify potential influencing factors. Notably, patients with HCV in Asian populations showed lower RBP4 levels compared to healthy individuals. We hypothesize that RBP4 levels may vary among different populations due to diverse factors, leading to heterogeneous expression levels. First, regional diversity in HCV genotypes may contribute to this variation. A comprehensive study encompassing 117 countries and 90% of the global population has highlighted the geographic variability in HCV genotype distribution [30]. Genotypes 1 and 3 are globally prevalent, while genotypes 2 and 4 are more prevalent in Africa, and genotypes 2 and 6 are common in Southeast Asia. The distinct pathogenic mechanisms of different HCV genotypes may influence RBP4 levels differently. Our results did not reveal a robust correlation between RBP4 levels and all forms of HCV infection, suggesting that RBP4 regulation may be specific to certain HCV genotypes. However, the scope of current studies is often limited to particular regions, with potential restrictions in genotype representation and a lack of focus on documenting these genotypes. This limitation hinders our ability to conclusively associate RBP4 levels with specific HCV genotypes. Therefore, future research should involve larger, multi-population studies that account for a range of HCV genotypes. Other factors, such as racial disparities, dietary habits, environmental factors, healthcare practices, and inconsistencies in testing methodologies and diagnostic criteria, may also influence RBP4 levels among HCV-infected populations. These factors deserve to be emphasized and controlled for in future studies.

We also investigated the potential association between RBP4 levels, metabolic parameters, and disease severity in patients with HCV. Contrary to studies reporting a significant correlation between insulin resistance and RBP4 levels [31,32], our study did not find a strong correlation between RBP4 and insulin resistance in patients with HCV. This discrepancy might be attributed to differences in the study populations. Previous studies focused on obese or diabetic cohorts, while our research concentrated on individuals with liver pathologies. Given the predominant role of the liver in producing RBP4, hepatic function can significantly impact RBP4 levels [33]. This interplay may result in a mitigated association between RBP4 levels and insulin resistance specifically within populations with liver disease, as suggested by the work of Fedders and Yagmur et al. [34,35]. A similar paradoxical finding was observed in lipid metabolism [36,37], where our study found no significant differences in RBP4 levels among patients with HCV with or without hepatic steatosis. This finding might also stem from the limited number of studies included in our analysis, which may affect the stability of the results.

Chayanupatkul et al. reported decreased serum RBP4 levels in patients with biliary atresia, which further diminished with advancing fibrosis [38]. This suggests the involvement of RBP4 in liver fibrosis pathogenesis. Similar inverse correlations between RBP4 levels and the severity of hepatic damage or fibrosis have also been observed in non-alcoholic fatty liver disease [39]. Considering liver fibrosis and liver function are reliable indicators of HCV disease progression [40], our meta-analysis examined the relationship between RBP4 levels and these factors. Consistent with prior research, our results revealed an inverse relationship between RBP4 levels and liver function in patients with HCV, with levels decreasing as fibrosis progresses. These findings position RBP4 as a potential novel non-invasive biomarker for assessing HCV severity, where reduced levels may indicate advanced hepatic impairment. Further research into the causal link between RBP4 and HCV progression could pave the way for innovative therapeutic strategies for the disease.

This study has several limitations. The scarcity of research on the RBP4-HCV association resulted in a limited number of included studies. Thus, our meta-analysis results should be interpreted cautiously. Furthermore, the significant heterogeneity across studies may impact the reliability of our results. Although we performed subgroup and sensitivity analyses to investigate potential sources, the reason for the high heterogeneity remained unexplained. Future research should aim for larger sample sizes and a broader spectrum of patient subgroups. The reliance on observational studies in this analysis also restricts our ability to infer causality between RBP4 levels and HCV disease progression or severity. Therefore, experimental studies, including in vivo and in vitro models, are needed to elucidate potential causal links between RBP4 levels and HCV infection.

## 5. Conclusions

In summary, our meta-analysis provides preliminary insights into the complex roles of RBP4 among patients with HCV. RBP4 levels appear to be influenced by a multitude of factors, highlighting the intricate nature of its metabolic regulation. Notably, a significant negative correlation between RBP4 levels and the severity of liver fibrosis, as well as liver function in patients with HCV, was observed. This correlation suggests that RBP4 could serve as an emerging non-invasive biomarker for assessing the severity of HCV infection. However, the current body of research on RBP4 is limited, and future, more directed studies are required to elucidate the relationship between RBP4 levels and HCV infection.

## Figures and Tables

**Table 1:** Main characteristics of the studies included in this meta-analysis.

1st Author (Year)	Country	Study Location	HCV Patients (*n*)	Health Control (*n*)	Study Design	Method of RBP4 Measurement	Quality Score	(Refs.)
Forrester (2009)	Spanish	Europe	43	108	Cross-sectional	ELISA	6	[19]
Huang (2009)	China	Asia	105	100	Cross-sectional	ELISA	6	[21]
Iwasa (2009)	Japan	Asia	81	19	Cross-sectional	ELISA	3	[20]
Petta (2011)	Italy	Europe	66	12	Cohort	ELISA	3	[16]
Kukla (2012)	Poland	Europe	75	41	Cross-sectional	ELISA	5	[23]
Chang (2020)	China	Asia	842	842	Cross-sectional	ELISA	6	[18]
Fayed (2020)	Egypt	Africa	50	20	Case-control	ELISA	7	[22]
Rasmy (2023)	Egypt	Africa	20	10	Cross-sectional	ELISA	4	[17]

Abbreviations: HCV, hepatitis C virus; RBP4, retinol-binding protein 4.

**Table 2:** Meta-analysis results of Fisher’s Z and the summary r between clinical parameters and RBP4 levels.

Clinical Parameters	Tests of Overall Effects of Meta-Analysis				Summary	
	Z	*p*	Fisher’s Z	95%CI	r	95% CI
HCV-RNA	0.58	0.56	−0.17	−0.73 to 0.40	−0.17	−0.62 to 0.28
Fibrosis	4.72	<0.01	−0.59	−0.84 to −0.35	−0.53	−0.69 to −0.37
HOMA-IR	0.63	0.53	−0.05	−0.21 to 0.11	−0.05	−0.21 to 0.11
ALT	4.11	<0.01	−0.32	−0.47 to −0.17	−0.31	−0.44 to −0.17
AST	3.71	<0.01	−0.29	−0.44 to −0.17	−0.29	−0.41 to −0.17

Abbreviations: r, correlation coefficients; RBP4, retinol-binding protein 4; CI, Confidence interval; HCV, hepatitis C virus; HOMA-IR, insulin resistance index; ALT, glutamic pyruvic transaminase; AST, aspartate transaminase.

## Data Availability

The data underlying this article are available in the article and in its online Supplementary Material.

## Supplementary Materials

The following supporting information can be downloaded at https://www.mdpi.com/article/10.3390/diseases12110291/s1: Figure S1: Publication bias: (A) funnel plot; (B) egger’s test. Figure S2: Duval and Tweedie trim-and-fill methods: (A) filled funnel plot; (B) filled forest plot.

## References

[1] Spearman C.W., Dusheiko G.M., Hellard M., Sonderup M. (2019). Hepatitis C. Lancet.

[2] Smith D.B., Bukh J., Kuiken C., Muerhoff A.S., Rice C.M., Stapleton J.T., Simmonds P. (2014). Expanded Classification of Hepatitis C Virus Into 7 Genotypes and 67 Subtypes: Updated Criteria and Genotype Assignment Web Resource. Hepatology.

[3] Syed G.H., Amako Y., Siddiqui A. (2010). Hepatitis C Virus Hijacks Host Lipid Metabolism. Trends Endocrinol. Metab..

[4] Parvaiz F., Manzoor S., Iqbal J., McRae S., Javed F., Ahmed Q.L., Waris G. (2014). Hepatitis C Virus Nonstructural Protein 5A Favors Upregulation of Gluconeogenic and Lipogenic Gene Expression Leading towards Insulin Resistance: A Metabolic Syndrome. Arch. Virol..

[5] Ramière C., Rodriguez J., Enache L.S., Lotteau V., André P., Diaz O. (2014). Activity of Hexokinase Is Increased by Its Interaction with Hepatitis C Virus Protein NS5A. J. Virol..

[6] Chang M.-L. (2016). Metabolic Alterations and Hepatitis C: From Bench to Bedside. World J. Gastroenterol..

[7] Sah U.K., Sah A.K., Ansari M., Chaudhary P., Gupta S., Kumar P., Sah J.P. (2023). HCV Co-Infection and Its Genotypic Distribution in HIV-Infected Patients in Nepalese Population. Trop. Med. Infect. Dis..

[8] van Dam R.M., Hu F.B. (2007). Lipocalins and Insulin Resistance: Etiological Role of Retinol-Binding Protein 4 and Lipocalin-2?. Clin. Chem..

[9] Blaner W.S. (1989). Retinol-Binding Protein: The Serum Transport Protein for Vitamin A*. Endocr. Rev..

[10] Tsutsumi C., Okuno M., Tannous L., Piantedosi R., Allan M., Goodman D.S., Blaner W.S. (1992). Retinoids and Retinoid-Binding Protein Expression in Rat Adipocytes. J. Biol. Chem..

[11] Yang Q., Graham T.E., Mody N., Preitner F., Peroni O.D., Zabolotny J.M., Kotani K., Quadro L., Kahn B.B. (2005). Serum Retinol Binding Protein 4 Contributes to Insulin Resistance in Obesity and Type 2 Diabetes. Nature.

[12] Jia W., Wu H., Bao Y., Wang C., Lu J., Zhu J., Xiang K. (2007). Association of Serum Retinol-Binding Protein 4 and Visceral Adiposity in Chinese Subjects with and without Type 2 Diabetes. J. Clin. Endocrinol. Metab..

[13] Tsutsumi T., Suzuki T., Shimoike T., Suzuki R., Moriya K., Shintani Y., Fujie H., Matsuura Y., Koike K., Miyamura T. (2002). Interaction of Hepatitis C Virus Core Protein with Retinoid X Receptor Alpha Modulates Its Transcriptional Activity. Hepatology.

[14] Lee H., Woo Y.-J., Kim S.S., Kim S.-H., Park B.-J., Choi D., Jang K.L. (2013). Hepatitis C Virus Core Protein Overcomes All-Trans Retinoic Acid-Induced Cell Growth Arrest by Inhibiting Retinoic Acid Receptor-Β2 Expression via DNA Methylation. Cancer Lett..

[15] Imran M., Waheed Y., Manzoor S., Bilal M., Ashraf W., Ali M., Ashraf M. (2012). Interaction of Hepatitis C Virus Proteins with Pattern Recognition Receptors. Virol. J..

[16] Petta S., Tripodo C., Grimaudo S., Cabibi D., Cammà C., Di Cristina A., Di Marco V., Di Vita G., Ingrao S., Mazzola A. (2011). High Liver RBP4 Protein Content Is Associated with Histological Features in Patients with Genotype 1 Chronic Hepatitis C and with Nonalcoholic Steatohepatitis. Dig. Liver Dis..

[17] Rasmy H.S., Elmalatawy M.A.E., ElKarmoty K.Z., Abdelwarth E.Y., Isaac A. (2023). Serum Retinol-Binding Protein 4 as a Predictor of Fibrosis Regression and Response to Direct-Acting Antiviral Drugs in Chronic Hepatitis C Virus Patients. Egypt. Liver J..

[18] Chang M.-L., Chen W.-T., Hu J.-H., Chen S.-C., Gu P.-W., Chien R.-N. (2020). Altering Retinol Binding Protein 4 Levels in Hepatitis C: Inflammation and Steatosis Matter. Virulence.

[19] Forrester J.E., McGovern B.H., Rhee M.S., Sterling R.K. (2009). The Individual and Combined Influence of HIV and Hepatitis C Virus on Dyslipidaemia in a High-Risk Hispanic Population. HIV Med..

[20] Iwasa M., Hara N., Miyachi H., Tanaka H., Takeo M., Fujita N., Kobayashi Y., Kojima Y., Kaito M., Takei Y. (2009). Patients Achieving Clearance of HCV with Interferon Therapy Recover from Decreased Retinol-Binding Protein 4 Levels. J. Viral Hepat..

[21] Huang J.-F., Dai C.-Y., Yu M.-L., Shin S.-J., Hsieh M.-Y., Huang C.-F., Lee L.-P., Lin K.-D., Lin Z.-Y., Chen S.-C. (2009). Serum Retinol-Binding Protein 4 Is Inversely Correlated with Disease Severity of Chronic Hepatitis C. J. Hepatol..

[22] Fayed H.M., Mahmoud H.S., Elaiw Mohamed Ali A. (2020). The Utility of Retinol-Binding Protein 4 in Predicting Liver Fibrosis in Chronic Hepatitis C Patients in Response to Direct-Acting Antivirals. Clin. Exp. Gastroenterol..

[23] Kukla M., Berdowska A., Stygar D., Gabriel A., Mazur W., Łogiewa-Bazger B., Sobala-Szczygieł B., Bułdak R.J., Rokitka M., Zajęcki W. (2012). Serum FGF21 and RBP4 Levels in Patients with Chronic Hepatitis C. Scand. J. Gastroenterol..

[24] Page M.J., McKenzie J.E., Bossuyt P.M., Boutron I., Hoffmann T.C., Mulrow C.D., Shamseer L., Tetzlaff J.M., Akl E.A., Brennan S.E. (2021). The PRISMA 2020 Statement: An Updated Guideline for Reporting Systematic Reviews. BMJ.

[25] Almirall J., Serra-Prat M., Bolíbar I., Balasso V. (2017). Risk Factors for Community-Acquired Pneumonia in Adults: A Systematic Review of Observational Studies. Respiration.

[26] Yao J.-M., Ying H.-Z., Zhang H.-H., Qiu F.-S., Wu J.-Q., Yu C.-H. (2023). Exosomal RBP4 Potentiated Hepatic Lipid Accumulation and Inflammation in High-Fat-Diet-Fed Mice by Promoting M1 Polarization of Kupffer Cells. Free Radic. Biol. Med..

[27] Frances L., Tavernier G., Viguerie N. (2021). Adipose-Derived Lipid-Binding Proteins: The Good, the Bad and the Metabolic Diseases. Int. J. Mol. Sci..

[28] Tacke F., Weiskirchen R., Trautwein C. (2008). Liver Function Critically Determines Serum Retinol-Binding Protein 4 (RBP4) Levels in Patients with Chronic Liver Disease and Cirrhosis. Hepatology.

[29] Mosnier J.F. (1996). Current Anatomico-Pathological Classification of Hepatitis: Characteristics of HCV Infection. Nephrol. Dial. Transpl..

[30] Messina J.P., Humphreys I., Flaxman A., Brown A., Cooke G.S., Pybus O.G., Barnes E. (2015). Global Distribution and Prevalence of Hepatitis C Virus Genotypes. Hepatology.

[31] Wolf G. (2007). Serum Retinol-Binding Protein: A Link between Obesity, Insulin Resistance, and Type 2 Diabetes. Nutr. Rev..

[32] Graham T.E., Yang Q., Blüher M., Hammarstedt A., Ciaraldi T.P., Henry R.R., Wason C.J., Oberbach A., Jansson P.-A., Smith U. (2006). Retinol-Binding Protein 4 and Insulin Resistance in Lean, Obese, and Diabetic Subjects. N. Engl. J. Med..

[33] Steinhoff J.S., Lass A., Schupp M. (2021). Biological Functions of RBP4 and Its Relevance for Human Diseases. Front. Physiol..

[34] Yagmur E., Weiskirchen R., Gressner A.M., Trautwein C., Tacke F. (2007). Insulin Resistance in Liver Cirrhosis Is Not Associated With Circulating Retinol-Binding Protein 4. Diabetes Care.

[35] Fedders R., Muenzner M., Weber P., Sommerfeld M., Knauer M., Kedziora S., Kast N., Heidenreich S., Raila J., Weger S. (2018). Liver-Secreted RBP4 Does Not Impair Glucose Homeostasis in Mice. J. Biol. Chem..

[36] Blaner W.S. (2019). Vitamin A Signaling and Homeostasis in Obesity, Diabetes, and Metabolic Disorders. Pharmacol. Ther..

[37] Hu R., Yang X., He X., Song G. (2023). The Relationship between NAFLD and Retinol-Binding Protein 4—An Updated Systematic Review and Meta-Analysis. Lipids Health Dis..

[38] Chayanupatkul M., Honsawek S., Chongsrisawat V., Vimolket L., Poovorawan Y. (2011). Serum Retinol Binding Protein 4 and Clinical Outcome in Postoperative Biliary Atresia. Hepatol. Int..

[39] Nobili V., Alkhouri N., Alisi A., Ottino S., Lopez R., Manco M., Feldstein A.E. (2009). Retinol-Binding Protein 4: A Promising Circulating Marker of Liver Damage in Pediatric Nonalcoholic Fatty Liver Disease. Clin. Gastroenterol. Hepatol..

[40] Zampino R., Marrone A., Restivo L., Guerrera B., Sellitto A., Rinaldi L., Romano C., Adinolfi L.E. (2013). Chronic HCV Infection and Inflammation: Clinical Impact on Hepatic and Extra-Hepatic Manifestations. World J. Hepatol..

