# Supplementary material for: Association Between Retinol-Binding Protein 4 Levels and Hepatitis C Virus Infection: A Meta-Analysis
Source: Diseases. 2024 Nov 13;12(11):291. doi: 10.3390/diseases12110291 (PMC11592848; doi:10.3390/diseases12110291)
Supplement: Supplementary file 1 [file diseases-12-00291-s001.zip › diseases-3130341-supplementary.pdf]

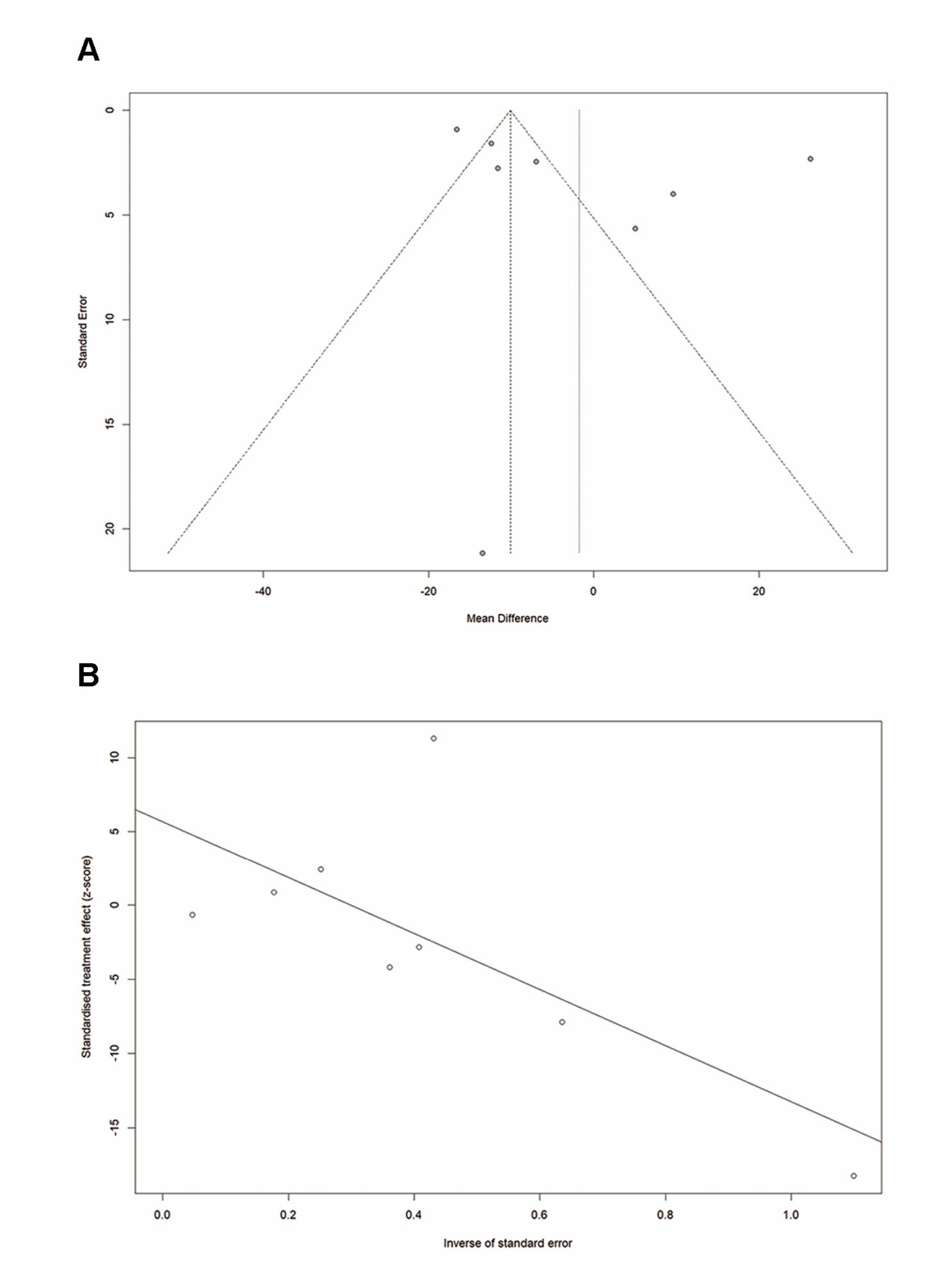


**Figure S1.** Publication bias. (**A**) Funnel plot. (**B**) Egger's test.

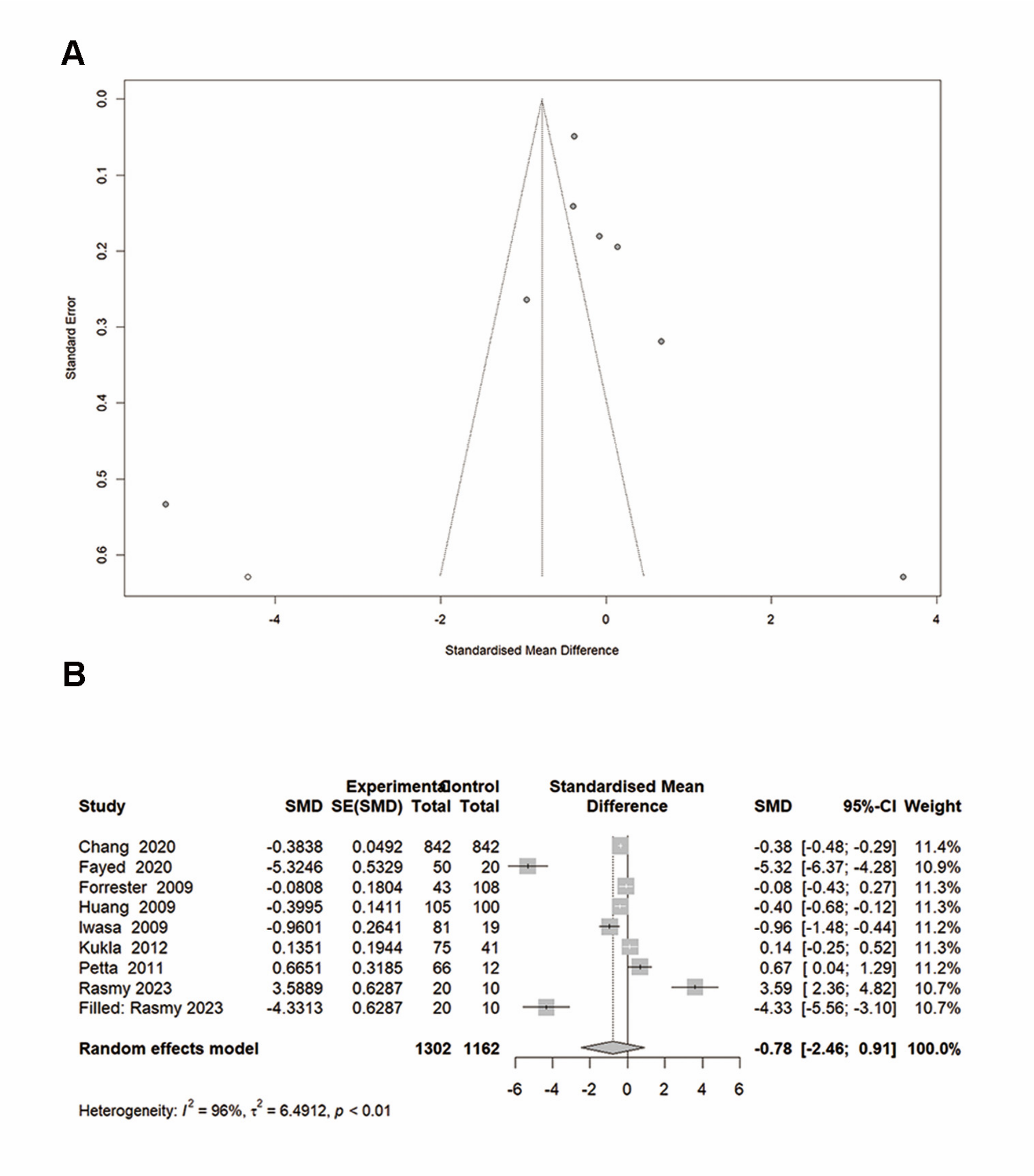


**Figure S2.** Duval and Tweedie trim-and-fill methods. (**A**) Filled funnel plot. (**B**) Filled forest plot.
